# Regenerative potential of allogeneic adipose tissue-derived mesenchymal cells in canine cutaneous wounds

**DOI:** 10.1186/s13028-020-0511-z

**Published:** 2020-03-12

**Authors:** Nathaly Enciso, Luis Avedillo, María Luisa Fermín, Cristina Fragío, Concepción Tejero

**Affiliations:** 1 Experimental Hematology UCM-Research Group, Veterinary Faculty, Complutense University of Madrid, Avda. Puerta de Hierro s/n, 28040 Madrid, Spain; 2 Department of Biochemistry and Molecular Biology, Veterinary Faculty, Complutense University of Madrid, Avda. Puerta de Hierro s/n, 28040 Madrid, Spain; 3 Department of Animal Surgery and Medicine, Veterinary Faculty, Complutense University of Madrid, Avda. Puerta de Hierro s/n, 28040 Madrid, Spain

**Keywords:** Adipose mesenchymal stem cells, Cutaneous wounds, Dog, Regenerative medicine

## Abstract

**Background:**

Mesenchymal stem cells (MSCs) have generated a great amount of interest over the past decade as a novel therapeutic treatment for a variety of diseases. Emerging studies have indicated that MSCs could enhance the repair of injured skin in canine cutaneous wounds.

**Case presentation:**

A healthy 2 years old Bodeguero Andaluz dog was presented with multiple skin bite wounds. Antibiotic and anti-inflammatory therapy was administered for 8 days. On day three, 10^7^ allogeneic adipose-derived mesenchymal stem cells (ASCs) were intradermally injected approximately equidistant to the ASCs treated wounds. Control wounds underwent conventional treatment with a topical antibacterial ointment until wound healing and closure. Wounds, skin morphology and healing progress were monitored via serial photographs and histopathology of biopsies obtained at day seven after ASC treatment. Histopathology revealed absence of inflammatory infiltrates and presence of multiple hair follicles in contrast to the non-ASCs treated control wounds indicating that ASC treatment promoted epidermal and dermal regeneration. ASCs were identified by flow cytometry and RT-PCR. The immunomodulatory role of ASCs was evidenced by coculturing peripheral blood mononuclear cells with allogeneic ASCs. Phytohemagglutinin was administered to stimulate lymphocyte proliferation. Cells were harvested and stained with an anticanine CD3-FITC antibody. The ASCs inhibited proliferation of T lymphocytes, which was quantified by reduction of carboxyfluorescein succinimidyl ester intensity using flow cytometry.

**Conclusions:**

Compared with conventional treatment, wounds treated with ASCs showed a higher regenerative capacity with earlier and faster closure in this dog.

## Background

Skin injuries are very common in veterinary medicine and emerging studies have indicated that mesenchymal stem cells (MSCs) could enhance repair of injured skin in canine cutaneous wounds [1–3]. Wound healing is a complex process comprising a cascade of molecular and cellular events involving multiple cell types, soluble factors, and matrices [4].

Recent advances in the field of cell therapy and regenerative medicine describe MSCs from different origins such as bone marrow, adipose tissue and umbilical cord-blood as potential biological products due to their self-renewal ability, immunosuppressive potential and ability to transdifferentiate [5]. MSCs are multipotent adult cells with immunomodulatory and regenerative properties; therefore, their therapeutic potential is being widely studied to evaluate their viability, safety and efficacy [6]. MSCs must have the capacity to differentiate and therefore they express stem cell and early differentiation markers [7].

In veterinary medicine, studies have focused on wound care with a main goal of accelerating the healing process. Thus, canine adult MSCs and tissue engineering technology have gained an important role in regenerative therapy in dogs with the potential as a research model for humans. Furthermore, MSCs show an immunomodulatory effect on the proliferation and apoptosis of allogeneic lymphocytes. These findings suggest that MSCs can be useful candidates for allogeneic cell therapy and transplantation without a major risk of rejection [8]. We hypothesize that application of cultured allogeneic adipose-derived mesenchymal stem cells (ASCs) is associated with more rapid repair of canine skin wounds compared to conventional wound treatment.

## Case report

**Fig. 1:**
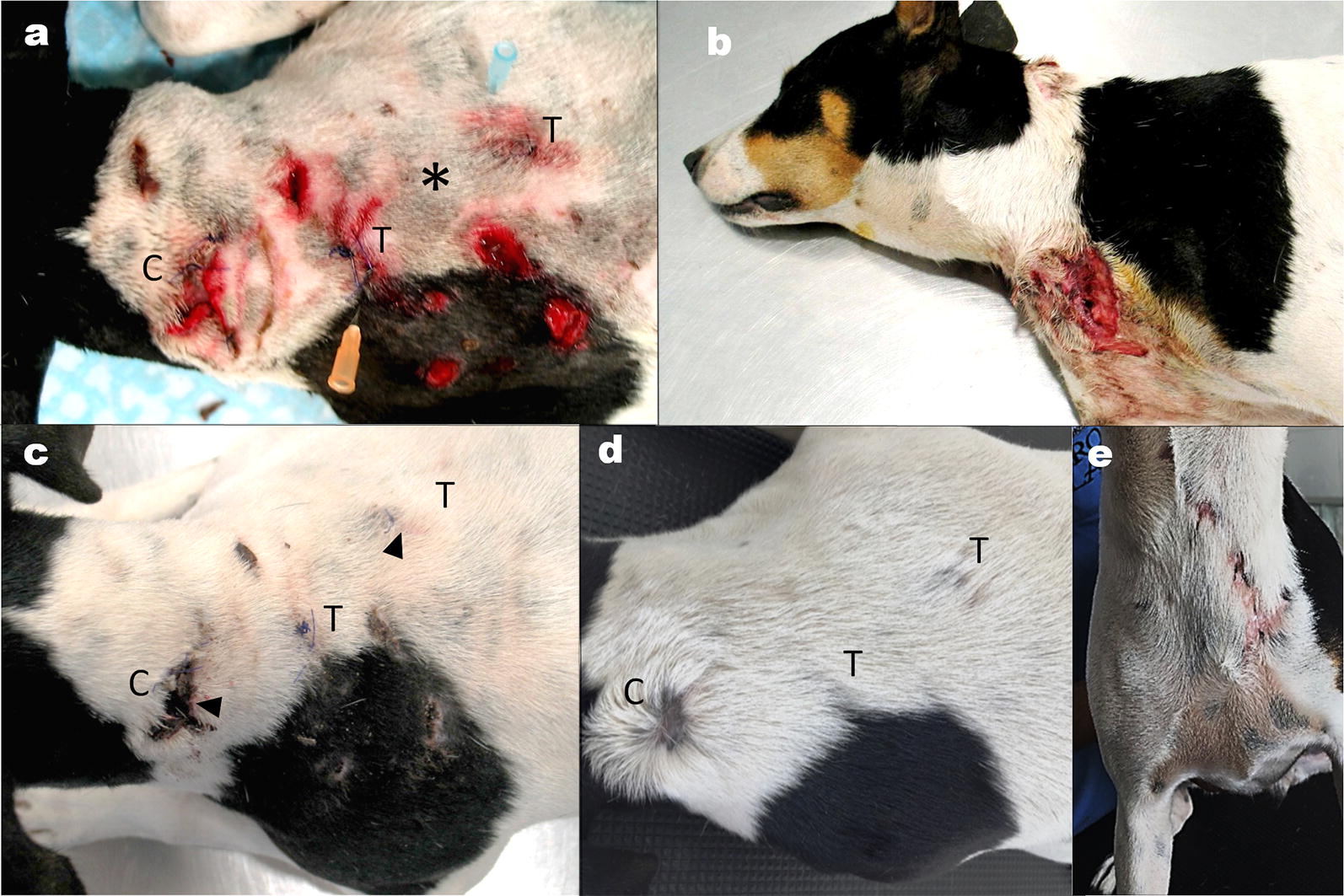
Healing process of wounds treated with allogeneic adipose-derived mesenchymal stem cells (ASCs) and conventional therapy. **a** Wounds on the neck (*C*: control) and dorsum (*T*: treated with ASCs) on day 1. Inoculation of ASCs was done equidistant from treated wounds (*asterisk*). **b** Non-treated deep wounds on chest on day 1. **c** Biopsy sites for treated and control wounds (*arrows head*). On day 7, treated wounds show faster healing than controls. **d** Treated wounds closed completely with re-occurrence of hair while the control wound remained hairless at day 25. **e** Chest wounds show multiple scars at day 25

The study was performed on a healthy 2-year-old female Bodeguero Andaluz dog with several deep skin bite wounds that were inflicted by another dog. The dog was treated according to the Spanish Real Decreto 53/2013 on animal welfare. All procedures were approved by the ethical committee of Veterinary Faculty from the Complutense University of Madrid (approval no. 08/2017), and the dog owner provided informed consent for the treatment. The deep wounds, i.e. wounds penetrating into the subcutis without muscular involvement, were localized on the chest, neck and dorsum (Fig. 1). Wounds on the dorsum were treated with ASCs while those on neck (used as control) and chest were treated conservatively. All wounds were partially sutured with conventional interrupted sutures with violet monofilament absorbable suture 2.0 (Atramat PDX. Mexico DF. Mexico). There were also superficial bite injuries penetrating the epidermis and the upper dermis adjacent to the skin wounds. These lesions were disinfected with chlorhexidine.

Initially, the dog was treated for 8 days with 0.2 mg/kg meloxicam (Metacam; Boehringer Ingelheim España, S.A, Barcelona, Spain) followed by 0.1 mg/kg q 24 h IM meloxicam, 15 mg/kg q 48 h IM amoxicillin trihydrate (Bivamox® L.A.; Boehringer Ingelheim España, S.A.), and 5 mg/kg q 24 h PO enrofloxacin (Ganadexil enroflocaxino; Industrial Veterinaria, S.A., Barcelona, Spain).

After three days with this treatment, 10^7^ allogeneic ASCs (see below) in phosphate buffered saline (PBS) as a vehicle were injected intradermally approximately equidistant (3 cm) to the margin of the treated wounds (Fig. 1a). Non ASC-treated wounds received conventional treatment with an ointment containing *Centella asiatica* extract and neomycin (Blastoestimulina® 1%; Almiral S.A., Barcelona, España) until the lesions were completely closed and re-epithelialized were observed, implying that healing was completed. Local immunoreactions or other adverse conditions did not develop after the allogeneic ASC injection or during the subsequent 25 days.

ASCs were obtained as previously described by Enciso et al. [9]. Briefly, omental adipose tissue was obtained from a healthy donor female dog undergoing elective sterilization, and ASCs were consecutively isolated and cultured. ASCs used for injection were cultured in Luria–Bertani medium (Scharlab S.L. Barcelona, Spain) to evaluate sterility. Before using the ASCs for therapy, cells were identified as MSCs according to the International Society for Cellular Therapy [10]. The cells were (1) CD90 and MHC I positive and CD45, CD34 and MHC II negative by flow cytometry, (2) *OCT4, RUNX2, SOX9* and *PPARɣ* genes were detected by reverse transcription polymerase chain reaction (RT-PCR) according to [9, 11], (3) they were able to adhere to plastic, and 4) had a fibroblast-like morphology.

**Fig. 2:**
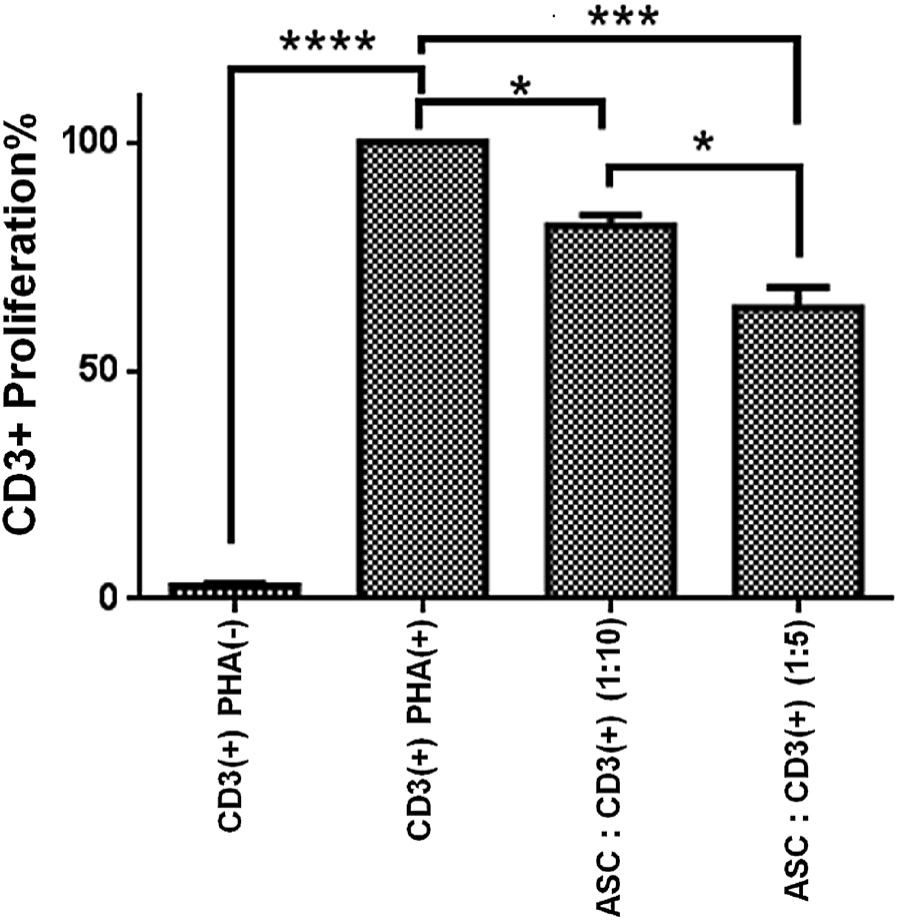
Immunomodulation. Inhibition of canine CD3+T cell proliferation cocultured with allogeneic adipose–derived mesenchymal stem cells (1:5 and 1:10 ratios) for 72 h and stimulated with phytohemagglutinin. Percentage of proliferation of phytohemagglutinin-stimulated lymphocytes. Values are mean ± SD. (*), (***), (****) indicate a statistically significant difference of P < 0.05, P < 0.001 and P < 0.0001, respectively

To examine the immunomodulatory potential of the ASCs, peripheral blood mononuclear cells (PBMCs) obtained from six healthy dogs were collected by Ficoll-Paque™ (GE Healthcare-Sigma, Saint Louis, MO, USA) according to the manufacturer’s protocol. Thereafter, the PBMCs were incubated with carboxyfluorescein succinimidyl ester (CFSE) (CellTrace™ Far Red, Invitrogen, Carlsbad, CA, USA) according to the manufacturer’s protocol. Stained PBMCs (5 × 10^5^ cells) were treated with phytohemagglutinin (PHA, 5 μg/mL; Sigma) to stimulate proliferation and cocultured with the allogeneic ASCs, previously incubated overnight at a ratio 1:5 (8 × 10^4^ cells/well) and 1:10 (4 × 10^4^ cells/well). After 72 h, cells were harvested and stained with anticanine CD3-FITC antibody (AbD-Serotec, Bio-Rad, Hercules, CA, USA). The ASCs were able to inhibit the proliferation of T lymphocytes, the response was studied by quantifying the reduction of CFSE intensity through cell divisions. In all experiments, unstimulated CD3( +) cells exhibited less than 2.7% proliferation. Proliferation induced by PHA on CD3( +) cells was considered to be 100%. ASCs inhibited proliferation of allogeneic CD3( +) cells at ratios of 1:10 (81%) and 1:5 (64%) (Fig. 2). This decrease was statistically significant in a ratio-dependent manner. The results were analysed using the software programs SPSS 25 (IBM Corporation, Endicott, NY, USA) and Graph Pad Prism version 6. All values are expressed as the mean ± SD. Normally distributed data were assessed using Kolmogorov–Smirnov and Shapiro–Wilk tests. Immunomodulatory potential was evaluated by comparing the percentage of PBMC proliferation between groups with an ANOVA post-Bonferroni t-test. Differences were considered significant when P < 0.05.

**Fig. 3:**
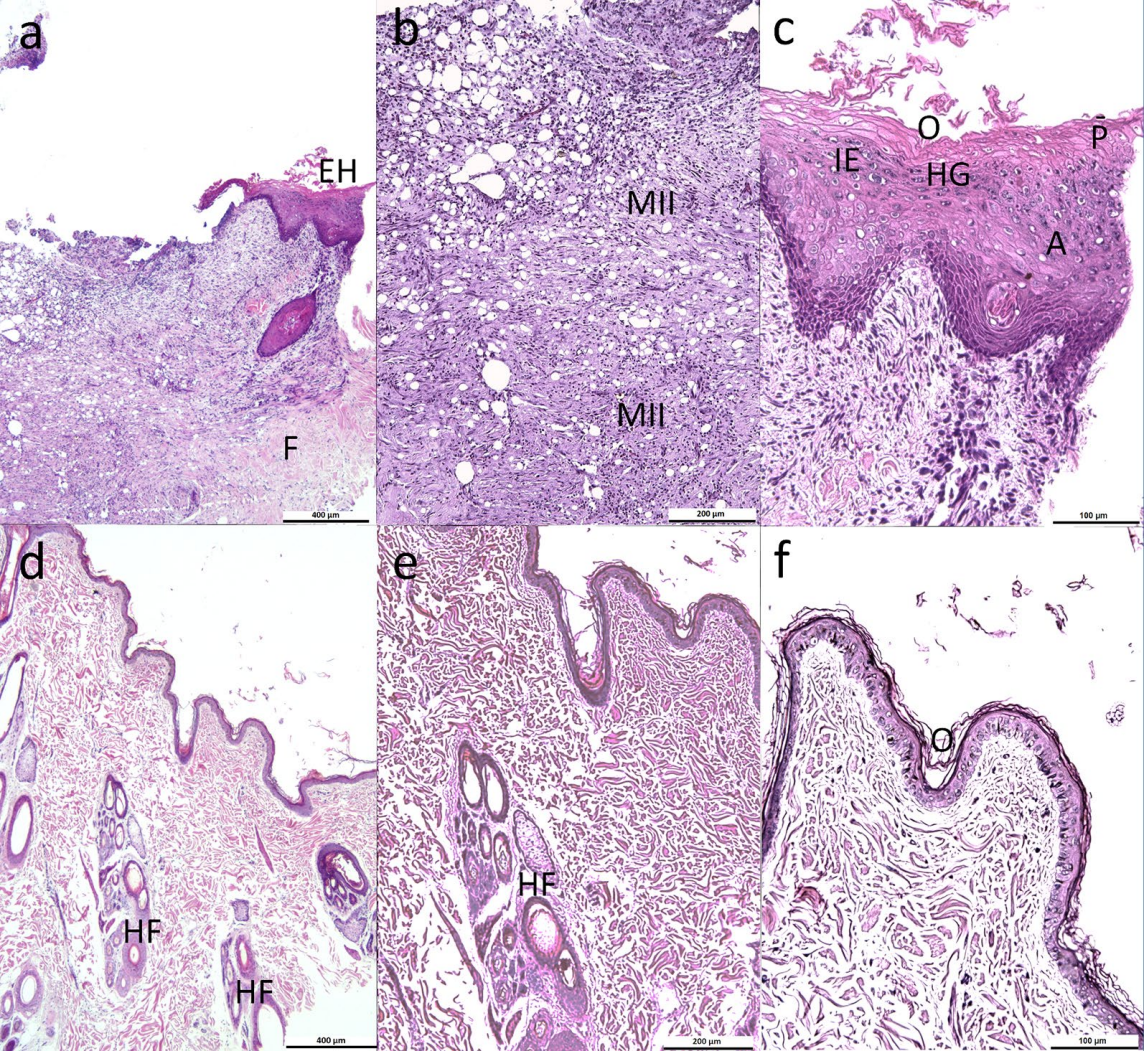
Histological sections of skin wounds biopsies after 7 days of treatment. **a**, **b**, and **c** Photomicrographs showing control wound; **d**, **e**, and **f** Photomicrographs showing wound treated with allogeneic adipose–derived mesenchymal stem cells. **a** Epidermal hyperplasia (*EH*) and full thickness fibroplasias (*F*) in dermis, bar: 400 µm**. b** Mononuclear inflammatory cell infiltrate (*MII*) extending mainly through the superficial and medium dermis, bar: 200 µm. **c** Orthokeratotic (*O*) and parakeratotic (*P*) hyperkeratosis, intracellular edema (*IE*), hypergranulosis (*HG*), and acanthosis (*A*), bar: 100 µm. The wound skin edge in control wound shows changes compatible with chronic dermatitis without follicular components. **d** and **e** Thin epidermis without evidence of inflammation; moreover multiple hair follicles (*HF*) can be observed, bar: 400 µm (d) and 200 µm (e). **f** Orthokeratosis (*O*), bar: 100 µm. Treated wound shows histological features compatible with the process of skin regeneration. **a**–**f** Hematoxylin and eosin staining

On day 7 after initiating ASC treatment, 4 mm diameter punch biopsies were taken from the edges of the control and the ASC treated wounds (Fig. 1c) for histopathology. The skin biopsies were formalin-fixed, paraffin-embedded, processed, sectioned at 3–5 µm and stained with hematoxylin and eosin (H&E). The epidermis of control wound (Fig. 3a–c) was characterized by epidermal hyperplasia, intracellular edema, hypergranulosis and ortho- and parakeratotic hyperkeratosis. The dermis had fibroplasia of all of its layers. We also observed the presence of a mononuclear inflammatory infiltrate extending mainly through the superficial and medium dermis. These findings were compatible with chronic dermatitis without follicular components, typical of a normal wound healing process [4]. The ASC treated skin wound (Fig. 3d–f) showed an almost normal skin with thin epidermis and orthokeratosis. There were no signs of dermal inflammation and multiple hair follicles in different stages of activity were present.

Re-epithelization was illustrated by photographs taken on the first day and at 7 and 25 days after ASC treatment (Fig. 1). On day 7, the treated deep wounds displayed accelerated skin closure compared with the control wound (Fig. 1c). On the other hand, on day 7 we also found unhealed lesions, probably because they were not sutured. On day 25, treated wounds and superficial skin lesions were completely closed and hair-bearing skin was observed (Fig. 1d). Nevertheless, the control wounds continued to exhibit hairless skin at that time. Figure 1b shows a deep non-treated wound at presentation and its appearance 25 days later (Fig. 1e) showing multiple hairless scars.

## Discussion and conclusions

In this case report, the role of allogeneic ASCs in wound healing therapy in a dog with multiple bite wounds was evaluated by comparison with lesions treated conservatively. Healing was more rapid in wounds treated with allogeneic ASCs thus indicating the potential of this therapy on enhancing wound healing. However, additional cases need to be studied before definitive conclusions can be drawn. It is important to note that the healing enhancement appeared as early as day 7 and the wounds were completely closed on day 25. In addition, in the ASC treated wounds and the adjacent superficial skin lesions (Fig. 1d), re-occurrence of hair was seen after 25 days, probably as a result of transmigration of the ASCs [12], while wounds in the neck (Fig. 1d) and chest (Fig. 1e) still were hairless. It is now well known that stem cells (mostly MSCs) can be recruited to participate in skin repair [13]. Experimental studies have shown the capability of MSCs in accelerating cutaneous wound repair both in chronic and acute wounds in dogs and humans [1, 14].

The presence of a regenerative process is also supported by histopathology findings, which revealed better re-epithelialization, reduced inflammatory infiltrate and presence of multiple hair follicles in different stages of activity on day 7 after treatment with ASCs. Conversely, biopsy of the non-treated wound revealed usual healing that was mainly characterized by an inflammation phase with a mononuclear infiltrate representing the standard healing process [15].

It is important to note that the use of allogeneic canine ASCs does not carry the risk of graft rejection. We demonstrated the immunomodulatory effect of ASCs suppressing the allogeneic response of lymphocytes. These data confirm the safety of allogeneic canine ASC therapy. These results are comparable to those reported by others [16, 17] and will serve as a useful tool for cell therapies and allogeneic stem cell transplantation between major histocompatibility complex (MHC)-incompatible recipients. This immunomodulatory effect is also supported by the fact that MSCs do not express MHC II [9].

To the best of our knowledge, our experimental treatment of one dog with bite wounds demonstrates for the first time the regenerative potential of allogeneic ASCs in canine cutaneous wounds. This treatment may represent a novel therapeutic approach in traumatic canine skin wounds, providing faster wound healing and re-epithelization.

## Data Availability

The datasets generated during and/or analyzed during the current study are available from the corresponding author on reasonable request.

## Abbreviations

ASCs: Adipose-derived mesenchymal stem cells
CFSE: Carboxyfluorescein succinimidyl ester
MHC: Major histocompatibility complex
MSCs: Mesenchymal stem cells
PBMC: Peripheral blood mononuclear cell
PHA: Phytohemagglutinin
